# Distinct Lineages of Bufavirus in Wild Shrews and Nonhuman Primates

**DOI:** 10.3201/eid2107.141969

**Published:** 2015-07

**Authors:** Michihito Sasaki, Yasuko Orba, Paulina D. Anindita, Akihiro Ishii, Keisuke Ueno, Bernard M. Hang’ombe, Aaron S. Mweene, Kimihito Ito, Hirofumi Sawa

**Affiliations:** Hokkaido University, Sapporo, Japan (M. Sasaki, Y. Orba, P.D. Anindita, A. Ishii, K. Ueno, K. Ito, H. Sawa);; University of Zambia, Lusaka, Zambia (B.M. Hang’ombe, A.S. Mweene)

**Keywords:** Parvovirus, animals, humans, molecular epidemiology, phylogeny, Zambia, viruses, shrews, wildlife, nonhuman primates

## Abstract

Viral metagenomic analysis identified a new parvovirus genome in the intestinal contents of wild shrews in Zambia. Related viruses were detected in spleen tissues from wild shrews and nonhuman primates. Phylogenetic analyses showed that these viruses are related to human bufaviruses, highlighting the presence and genetic diversity of bufaviruses in wildlife.

Bufavirus (BuV), a recently described parvovirus, was initially discovered in the feces of a child with diarrhea in Burkina Faso in 2012 (*1*). Thereafter, BuV was identified in fecal samples from children and adults with gastroenteritis in Bhutan, Finland, and the Netherlands in 2014 (*2**–**4*), respectively. Genome sequences and phylogenetic analyses revealed that BuV comprised at least 3 genotypes and was distinct from all other known members of the *Parvoviridae* family (*1**,**2*). The International Committee on Taxonomy of Viruses assigned BuV as a new species of the genus *Protoparvovirus* in the subfamily *Parvovirinae* (*5*). Whether BuV is an etiologic agent of human gastroenteritis remains unclear, but knowledge about its distribution and genetic divergence in humans is accumulating. However, whether BuV infection exists in wildlife remains unanswered. Through use of metagenomics, we previously described the enteric virome of wild shrews of the *Crocidura* genus sampled at Mpulungu, Zambia, in 2012 (*6*). From this sequence dataset (GenBank/EMBL/DDBJ accession no. DRA002561), we identified sequence reads related to BuV. Here, we describe the genome of this new parvovirus.

## The Study

We determined the nearly complete genome sequence of BuV, which we named Mpulungu BuV (MpBuV), by filling genome gaps with primer walking and rapid amplification of cDNA ends (GenBank/EMBL/DDBJ accession no. AB937988). The MpBuV genome comprises 4,613 nt and encodes open reading frames of the nonstructural protein (NS) 1 and the viral capsid protein (VP) 1 and VP2 (Technical Appendix Figure). blastp (http://blast.ncbi.nlm.nih.gov) searches showed that the MpBuV NS1, VP1, and VP2 proteins were closely related to those of human BuVs and the WUHARV parvovirus (E-value = 0.0). WUHARV parvovirus, identified in rhesus monkeys experimentally infected with simian immunodeficiency virus under laboratory conditions in the United States, was found to be a closely related to human BuVs (*7*).

In MpBuV, NS1 shares 52.5% aa identity with NS1 of human BuV (GenBank accession no. JX027296). We found that the amino acid sequence identity of VP1 between MpBuV and human BuV is 52.3%, whereas that of VP2 is 51.4%. Similar to human BuV, MpBuV showed potential splicing signals in the VP1 coding region. We also identified the parvovirus-conserved amino acid motifs in NS1, VP1, and VP2 of MpBuV (Technical Appendix Figure) (*8**–**12*). Phylogenetic analysis was performed as described previously (*6*). A Bayesian phylogenetic tree was generated on the basis of the full-length NS1 proteins of MpBuV, human BuVs, and representative parvoviruses. MpBuV clustered with human BuVs and WUHARV parvovirus (Technical Appendix Figure). According to the species demarcation criteria of the International Committee on Taxonomy of Viruses, each parvovirus species encodes an NS1 protein sharing <85% aa sequence identity with other known parvovirus species (*5*). The NS1 protein of MpBuV exhibited <58% sequence identity with that of any known parvovirus species; therefore, we propose that MpBuV is a new species within the *Protoparvovirus* genus.

Next, we performed PCR screening for MpBuV on shrews captured in Mpulungu. DNA was extracted from intestinal content suspensions and tissue specimens by using a High Pure Viral Nucleic Acid Kit (Roche Diagnostics, Mannheim, Germany) and QIAamp DNA Mini kit (QIAGEN, Hilden, Germany), respectively. PCR was performed by using Tks Gflex DNA polymerase (TAKARA BIO, Otsu, Japan), forward primer MpBuV-F1 (position 2739–2763 in MpBuV genome, 5′-GAAGTGGTGTTGGTCATTCTACTGG-3′) and reverse primer MpBuV-R1 (position 3523–3546, 5′-GTTGGAGGTACACATGGATGAGGA-3′). We detected the MpBuV genome in 5 (22%) of the 23 samples from the intestinal contents of individual shrews captured in Mpulungu. We then tested for the presence of MpBuV in the lung, spleen, liver, and kidney tissues of the shrews that were PCR positive for MpBuV by screening of intestinal contents. The MpBuV genome was detected in 5 spleen and 4 liver samples from the 5 shrews.

This discovery of MpBuV urged us to further investigate BuVs and related parvoviruses in wildlife. We designed degenerate primers for nested PCR screening on the basis of a multiple sequence alignment of the NS1 gene from human BuV, WUHARV parvovirus and MpBuV as follows: BuV-F1 (position 190–215 in MpBuV genome, 5′-TCAAWRTMACCTGGAAAGACTACAGA-3′) and BuV-R1 (position 1503–1534, 5′-TCATTGGTTGTCATKAYWACTGGAGTTGGTTC-3′) for the first PCR round, and BuV-F2 (position 980–1006, containing an equimolar mixture of 5′-AGAAAAATGGATGCTCCAAGATCCAGA-3′ and 5′-AGAAAAATGGATGCTTGGTGAWCCWGA-3′) and BuV-R2 (position 1444–1465, 5′-ATTGCTTGGCCACTCATGATKG-3′) for the second PCR round. PCRs were performed by using Tks Gflex DNA polymerase and the annealing temperatures were set at 50°C and 55°C for the first and second PCR round, respectively. 

We screened 536 spleen tissue specimens from wildlife. The specimens from 3 nonhuman primate species, 2 shrew species, and 14 rodent genera from 6 locations in Zambia were used for different research projects, as reported previously (*13**,**14*). All our sampling activities were conducted with the permission of the Zambia Wildlife Authority (Act No. 12 of 1998). We chose spleen tissue for nested PCR screening because specimens of intestinal contents were unavailable for almost all of the animals we sampled. As we described, MpBuV was detected in spleen samples in addition to samples of intestinal contents from the same animals.

Nested PCR screening for BuVs detected MpBuV from 5 shrews in Mpulungu; these were the same animals that were PCR-positive for MpBuV by using a specific primer set targeting MpBuV. Nested PCR was also positive in 3 primates and 12 shrews (Table). The PCR amplicons with expected size (≈480 bp) were subjected to direct sequence analysis. A Bayesian phylogenetic analysis was performed as described by using the partial nucleotide sequences obtained for the NS1 gene with the exception of the primer sequences (434–440 bp) and the corresponding genome regions of known protoparvoviruses and amdoviruses. The BuVs detected clustered with human BuVs and the WUHARV parvovirus and are distinguishable from other protoparvoviruses (Figure). The BuVs detected can be divided into MpBuV and 3 other strains; we have tentatively named these Solwezi BuV, Mfuwe BuV, and Livingstone BuV (Figure). Solwezi BuV, which shares 79% nt sequence identity with MpBuV, was identified in 12 shrews (*Crocidura luna*) in Solwezi. Mfuwe BuV and Livingstone BuV were identified in 2 yellow baboons (*Papio cynocephalus*) in Mfuwe and a chacma baboon (*P. ursinus*) in Livingstone, respectively. Both baboon-derived BuVs were closely related to the WUHARV parvovirus. These results indicate the presence of BuVs in wild nonhuman primates and in wild shrews.

**Table:** Sample information and nested PCR screening results for bufavirus, Zambia

Animal,* species (common name)	Location	Year	PCR positive/total
Primate			
*Papio cynocephalus* (yellow baboon)	Mfuwe	2009	2/50
*P. ursinus* (Chacma baboon)	Livingstone	2010–2011	1/50
*Chlorocebus pygerythrus* (vervet monkey)	Mfuwe	2009	0/50
	Livingstone	2010–2011	0/39
Shrew			
*Crocidura hirta* (lesser red musk shrew)	Livingstone	2011	0/2
	Mpulungu	2012	5/22
	Namwala	2012	0/2
	Mazabuka	2013	0/4
	Solwezi	2013	0/2
*C. luna* (moonshine shrew)	Mpulungu	2012	0/1
	Solwezi	2013	12/16
Rodent			
*Mastomys natalensis* (African soft-furred rat)	Livingstone	2011	0/35
	Mpulungu	2012	0/28
	Namwala	2012	0/29
	Mazabuka	2013	0/57
	Solwezi	2013	0/56
Other species†	Livingstone	2011	0/9
	Mpulungu	2012	0/20
	Namwala	2012	0/34
	Mazabuka	2013	0/16
	Solwezi	2013	0/14
Total			20/536

*For this analysis, the template DNA was prepared from the spleen tissues of the animals indicated. †Other species from the genera.*Aethomys, Arvicanthis, Cricetomys, Gerbilliscus, Grammomys, Graphiurus, Lemniscomys, Mus, Paraxerus, Pelomys, Rattus, Saccostomus*, and *Steatomys*.

**Figure:**
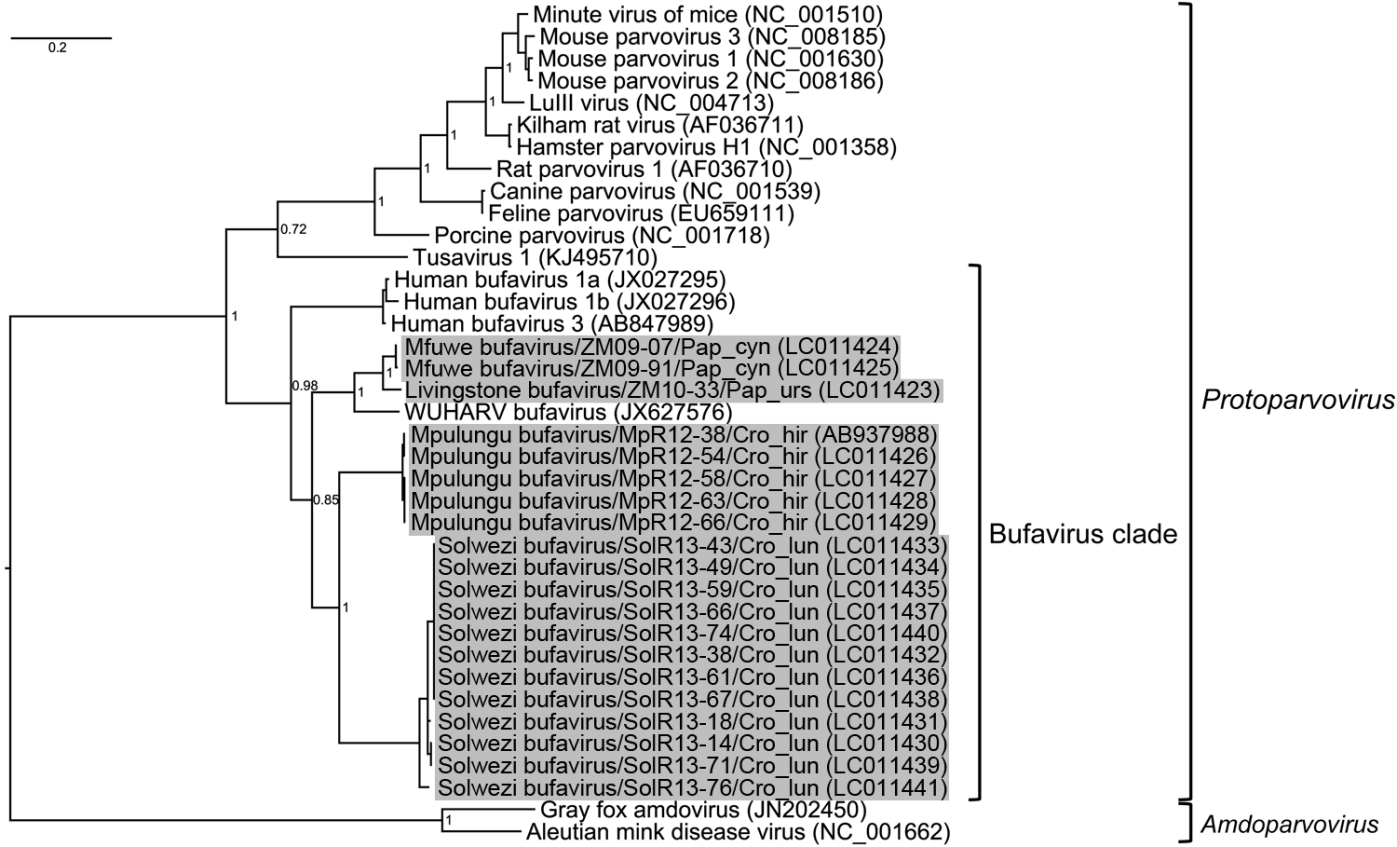
Partial nonstructural protein (NS) 1 gene phylogeny of newly identified bufaviruses, Zambia. The Bayesian phylogenetic tree was generated by using the partial NS1 gene fragments (434–440 bp) of bufaviruses and the corresponding region of known protoparvoviruses and amdoviruses. Gray shading indicates bufaviruses identified in this study. GenBank accession numbers of viral sequences are shown in parentheses. Bayesian posterior probabilities are indicated at each tree root. Scale bar indicates nucleotide substitutions per site.

## Conclusions

The nearly complete genome sequence of a new parvovirus, MpBuV, was obtained from a wild shrew in this study. blastp searches indicated that each MpBuV open reading frame shared the highest amino acid identity with other known BuVs. Furthermore, our phylogenetic analysis showed that MpBuV clustered with BuVs but was distinct from any other known parvovirus. Accordingly, we propose that MpBuV should be considered a new species of BuV.

Our nested PCR screening identified 3 additional BuV strains: Solwezi BuV, Mfuwe BuV, and Livingstone BuV. These protoparvoviruses are also phylogenetically related to known BuVs and derived from wildlife (i.e., shrews and nonhuman primates). These results show the presence of human BuV-related genomes in wildlife expanding our knowledge of the distribution and genetic diversity of BuVs.

In summary, we investigated the situation regarding BuVs in Zambian wildlife. Thus far, no evidence exists of BuV transmission between humans and wildlife. Our nested PCR should be helpful for detecting BuVs in mammals and lead to better understanding of the distribution of BuVs.

Technical Appendix Genome organization and phylogeny of Mpulungu bufavirus.
