# Supplementary material for: Distinct Lineages of Bufavirus in Wild Shrews and Nonhuman Primates
Source: Emerg Infect Dis. 2015 Jul;21(7):1230–3. doi: 10.3201/eid2107.141969 (PMC4480391; doi:10.3201/eid2107.141969)
Supplement: Technical Appendix — Genome organization and phylogeny of Mpulungu bufavirus. [file 14-1969-Techapp-s1.pdf]

Article DOI: http://dx.doi.org/10.3201/eid2107.141969

Distinct Lineages of Bufavirus in Wild Shrews and Nonhuman Primates

Technical Appendix

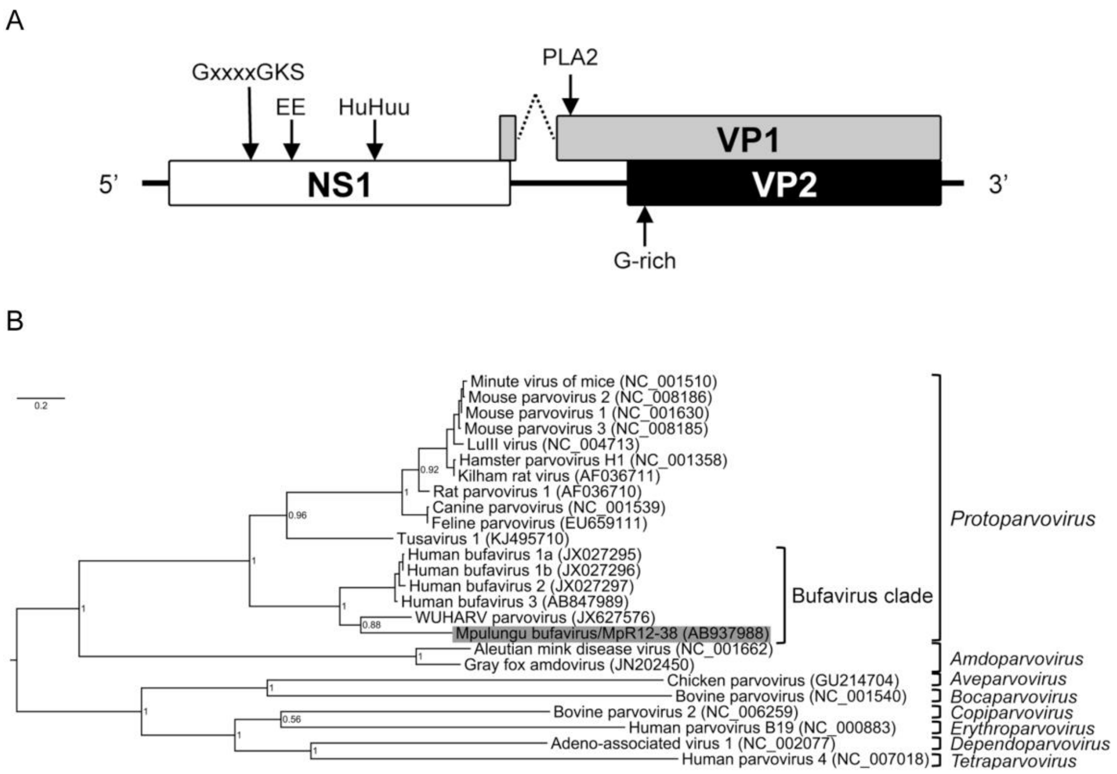

Technical Appendix Figure. Genome organization and phylogeny of Mpulungu bufavirus. A) Diagram of the predicted genome organization of Mpulungu bufavirus. The positions of the conserved helicase Walker A (GxxxxGKS), Walker B (EE), and replication initiator motifs (HuHuu) in NS1 and the phospholipase A2 (PLA2) and glycine-rich region (G-rich) in VP1 and VP2 are shown. B) The Bayesian phylogenetic tree was generated using the deduced amino acid sequences of the full-length NS1 of Mpulungu bufavirus and representative parvoviruses. The Mpulungu bufavirus is shaded in gray. GenBank accession numbers of the parvovirus sequences are shown in parentheses. Bayesian posterior probabilities are indicated at each tree root. NS, nonstructural protein; VP, viral capsid proteins. Scale bar represents 0.2 substitutions per site.
